# CAGW Peptide Modified Biodegradable Cationic Copolymer for Effective Gene Delivery

**DOI:** 10.3390/polym9050158

**Published:** 2017-04-28

**Authors:** Xinghong Duo, Jun Wang, Qian Li, Agnaldo Luis Neve, Mary Akpanyung, Abdelilah Nejjari, Zaidi Syed Saqib Ali, Yakai Feng, Wencheng Zhang, Changcan Shi

**Affiliations:** 1 School of Chemical Engineering and Technology, Tianjin University, Yaguan Road 135, Tianjin 300350, China; wlkdxh@163.com (X.D.); wangdajuner@163.com (J.W.); liqian200512@126.com (Q.L.); agnevesh3@yahoo.com (A.L.N.); splendymary@yahoo.com (M.A.); abdelilahnejjari23011991@gmail.com (A.N.); saqib_48@outlook.com (Z.S.S.A.); 2 School of Chemistry and Chemical Engineering, Qinghai University for Nationalities, Xining 810007, Qinghai, China; 3 Collaborative Innovation Center of Chemical Science and Chemical Engineering (Tianjin), Weijin Road 92, Tianjin 300072, China; 4 Joint Laboratory for Biomaterials and Regenerative Medicine, Tianjin University-Helmholtz-Zentrum Geesthacht, Yaguan Road 135, Tianjin 300350, China; 5 Key Laboratory of Systems Bioengineering of Ministry of Education, Tianjin University, Yaguan Road 135, Tianjin 300350, China; 6 Department of Physiology and Pathophysiology, Logistics University of Chinese People′s Armed Police Force, Tianjin 300162, China; wenchengzhang@yahoo.com; 7 Institute of Biomaterials and Engineering, Wenzhou Medical University, Wenzhou 325011, Zhejiang, China; 8 Wenzhou Institute of Biomaterials and Engineering, Ningbo Institute of Industrial Technology, Chinese Academy of Sciences, Wenzhou 325011, Zhejiang, China

**Keywords:** PLGA-*g*-PEI, polyethylenimine, endothelial cells, CAGW peptide, gene carrier, transfection

## Abstract

In recent years, gene therapy has become a promising technology to enhance endothelialization of artificial vascular grafts. The ideal gene therapy requires a gene carrier with low cytotoxicity and high transfection efficiency. In this paper, we prepared a biodegradable cationic copolymer poly(d,l-lactide-*co*-glycolide)-graft-PEI (PLGA-*g*-PEI), grafted Cys-Ala-Gly-Trp (CAGW) peptide onto this copolymer via the thiol-ene Click-reaction, and then prepared micelles by a self-assembly method. pEGFP-ZNF580 plasmids (pDNA) were condensed by these micelles via electrostatic interaction to form gene complexes. The CAGW peptide enables these gene complexes with special recognition for endothelial cells, which could enhance their transfection. As a gene carrier system, the PLGA-*g*-PEI-*g*-CAGW/pDNA gene complexes were evaluated and the results showed that they had suitable diameter and zeta potential for cellular uptake, and exhibited low cytotoxicity and high transfection efficiency for EA.hy926 cells.

## 1. Introduction

Coronary bypass surgery is one of the most important therapeutic methods for atherosclerotic vascular diseases [1,2]. However, the failure of artificial blood vessels after implantation cannot be avoided because of the formation of thrombus caused by their low hemocompatibility. Many strategies have been explored to improve the hemocompatibility and rapid endothelialization of artificial blood vessels [3]. Hydrophilic modification has been proven to be an effective method [4,5]. Lendlein et al. grafted different molecular weight and different terminal groups of poly(ethylene glycol) onto the surface of poly(ether amide) material [6]. This surface modification could reduce the adsorption of bovine serum albumin, but the adsorption effect of fibrinogen was not changed. Biological active antibodies were linked on the surface of the artificial blood vessel. The antibody could specifically bind to endothelial cells (ECs) via surface antigens in peripheral blood, thus promote EC adhesion and proliferation, accelerate endothelialization, and reduce thrombus formation and endometeium hyperplasia.

Gene therapy serves as a promising therapeutic method, and has become a hot topic [7,8,9]. The key issue in gene therapy is to develop highly efficient and safe gene carriers to transfer DNA or RNA to specific cells [10]. As the “gold” standard non-viral gene carrier, polyethylenimine (PEI) 25 kDa is widely used as an effective gene delivery system in vitro, because of its high transfection efficiency [11]. Generally, PEI with high molecular weight exhibits high transfection efficiency, but its cytotoxicity is also high [12,13,14]. In order to overcome this shortcoming, many researchers have modified low molecular weight PEI to enable its gene delivery function and low cytotoxicity. Poly(ethylene glycol) (PEG) modification is a useful method to decrease PEI’s cytotoxicity [15]. Zaric et al. prepared the glucose derivative modified PEI with low cytotoxicity [16]. In addition, Wang et al. prepared DNA complexes using PEI-Cholesterol 1.8 kDa and 10 kDa at a N/P ratio of 15/1. The transfection of these DNA complexes in Jurkat cells showed high levels of expressed green fluorescent protein (GFP) with low toxicity [17]. Nam et al. found that PEI–PLGA aggregates were readily adsorbed onto cell surfaces and translocated into the cytoplasm [18]. In addition, many biodegradable polymeric nanoparticles (NPs) have been used in gene delivery due to their biodegradability [19,20,21,22]. 

Short synthetic peptides, such as Arg-Gly-Asp (RGD) [22,23,24], Arg-Glu-Asp-Val (REDV) [25,26,27,28,29,30], and Cys-Ala-Gly (CAG) [31,32], can adhere to ECs and exhibit advantages for rapid endothelialization of biomaterial surfaces [33,34]. The RGD peptide has been widely used as a cell identification site [35,36]. However, it cannot selectively adhere ECs over other cells. In our previous study, we reported actively targeting NPs for ECs by using biodegradable polymers linking the Arg-Glu-Asp-Val (REDV) functional peptide. These polymers could condense pEGFP-ZNF580 (pZNF580 or pDNA) to form NPs/pZNF580 gene complexes [2,28,29,30]. These complexes showed low cytotoxicity and excellent activity targeting function for ECs [10,25,27,29]. They could enhance the proliferation and migration of ECs in vitro [30,37]. Alternatively, tripeptide Cys-Ala-Gly (CAG) has also attracted more attention. This peptide possesses high selectivity for ECs over smooth muscle cells (SMCs), and can specifically adhere to ECs [38,39]. In our previous work, an anti-thrombogenic platform demonstrated the associational effect of EC-specific adhesion (CAG peptide) and anti-nonspecific adhesion (PEG component) [31]. We also linked the CAG peptide on gene carriers via hetero-functional NHS-PEG-OPSS and formed a core-shell-corona structure. Owing to the function of the CAG peptide, the transfection efficiency had been strongly enhanced [32].

**Scheme 1:**
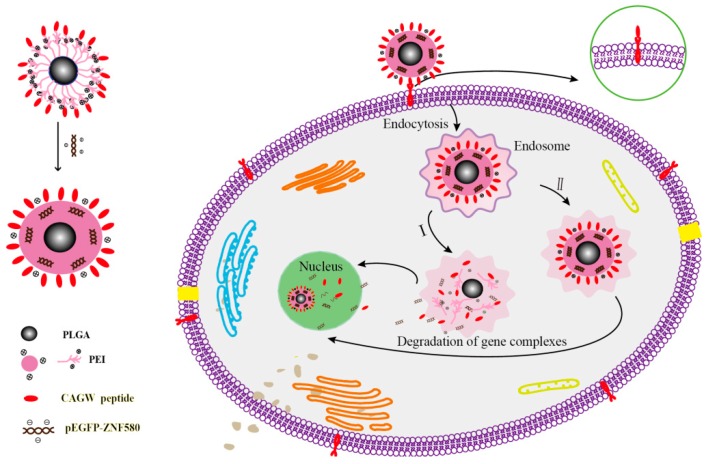
The formation of PLGA-*g*-PEI-*g*-CAGW/pDNA gene complexes, and the intracellular movement processes through active targeting adhesion mediated by gene complexes. After lysosome escape, gene complexes or pDNA could enter into the cell nucleus through path I or II.

Star-shaped polymers have low cytotoxicity and high transfection efficiency compared with linear polymers [40,41,42]. In this study, we prepared a biodegradable cationic copolymer poly(lactide-*co*-glycolide)-*graft*-PEI (PLGA-*g*-PEI). In order to endow this copolymer with the selective recognition of ECs, the CAGW peptide was used to modify this cationic copolymer. Then this copolymer was self-assembled to form micelles. CAGW peptide was preferentially located in the micellar outer layer (Scheme 1). pDNA was condensed and loaded onto these micelles through electrostatic interaction. Their size and zeta potential were determined by dynamic light scattering (DLS). The condensation ability of pDNA was studied by the agarose gel retardation assay. The in vitro cytotoxicity of gene complexes against EA.hy926 cells was evaluated by the thiazolyl blue assay (MTT assay). The transfection efficiency, gene expression, and cell migration were evaluated by transfection, Western blot assay, and wound healing assay.

## 2. Material and Methods

### 2.1. Materials 

Sorbitol, branched PEI (*M*_w_ = 10 kDa), stannous octoate (Sn(Oct)_2_), *N*-hydroxy succinimide (NHS), 1-ethyl-3-(3-dimethylaminopropyl)-carbodiimide hydrochloride (EDC), 4-dimethylamino pyridine (DMAP), and diallyl-carbamyl chloride were purchased from Sigma-Aldrich (St. Louis, MO, USA). d,l-Lactide (d,l-LA) and glycolide (GA) were obtained from Foryou Medical Device Co., Ltd. (Huizhou, China). Succinic anhydride, triethylamine (Et_3_N), 1,4-dioxane, toluene, 1-methyl-2-pyrrolidinone (NMP), and dimethyl sulfoxide (DMSO) were purchased from the Institute of Jiangtian Chemical (Tianjin, China) and were dried by refluxing over CaH_2_ and were then distilled. The EA.hy926 (human umbilical vein endothelial cells) cell line, which was used as an in vitro model, was purchased from the American Type Culture Collection (ATCC; Manassas, VA, USA), and EA.hy926 cells were cultured by the same method as our previous work [12,25]. Human umbilical artery smooth muscle cells (HUASMCs) and pEGFP-ZNF580 were preserved by the Department of Physiology and Pathophysiology, Logistics University of the Chinese People’s Armed Police Force. NMR spectrometer (AV-400, Bruker, Karlsruhe, Germany). Gel permeation chromatograph (GPC, Waters Company, Milford, CT, USA). Transmission Electron Microscopy JEM-2100F (TEM JEM-2100F, Japan Electronics, Tokyo, Japan).

### 2.2. Synthesis of Cationic Copolymer PLGA-g-PEI 

The synthesis of PLGA-*g*-PEI was performed according to a previously reported method [12]. Firstly, terminal hydroxyl copolymer poly(d,l-lactide-*co*-glycolide)-OH (PLGA-OH) was synthesized by ring-opening polymerization (ROP) of d,l-LA (4.5341 g, 31.49 mmol) and GA (0.5070 g, 4.37 mmol) initiated by the hydroxyl group of sorbitol (0.025 g, 0.14 mmol), using Sn(Oct)_2_ (0.4 mL, 0.25 mol/L) as a catalyst in a flame-dried and nitrogen-purged Schlenk flask. The Schlenk flask was sealed under dry nitrogen and immersed in an oil bath at 125 °C for 24 h. Then, the polymer was dissolved in chloroform and precipitated with ice-cold hexane, dried under vacuum at 25 °C until a constant weight.

Secondly, terminal carboxyl copolymer PLGA-COOH was synthesized by esterification with succinic anhydride. PLGA-OH (2.0 g, 0.06 mmol) was dissolved in 20 mL dioxane, then triethylamine (150 μL), DMAP (0.43 g, 3.5 mmol), and succinic anhydrides (0.34 g, 3.35 mmol) were added into a Schlenk flask. The flask was sealed and maintained at 25 °C for 24 h. The resulting copolymer re-dissolved in dichloromethane. The dichloromethane solution was extracted with 1 mol/L HCl solution, saturated NaCl solution, and saturated NaHCO_3_ solution. The organic phase was dried with anhydrous Na_2_SO_4_, then filtered and followed by rotary evaporation. The product PLGA-COOH was dried under vacuum at 25 °C until a constant weight.

Thirdly, PLGA-COOH (0.15 g, 0.0042 mmol) was dissolved in 10 mL DMSO in a Schlenk flask. EDC (0.0235 g, 0.1225 mmol) and NHS (0.0142 g, 0.1233 mmol) were added into this Schlenk flask, and stirred at 25 °C for 2 h. Then PEI (10 kDa, 0.162 g, 0.0162 mmol) DMSO solution was added and reacted at 25 °C for 24 h under nitrogen atmosphere. The reaction mixture was subjected to dialysis (molecular-weight cutoff (MWCO = 14 kDa)) for 48 h in order to remove DMSO and unreacted PEI.

### 2.3. Characterization of Cationic Copolymer PLGA-g-PEI

The ^1^H NMR, size, and zeta potential measurements were performed to characterize the PLGA-*g*-PEI. Average molecular weight (*M*_n_, *M*_w_) and molecular weight polydispersity index (PDI = *M*_w_/*M*_n_) of the copolymer were determined by gel permeation chromatography (GPC) using the same test conditions as in our previous work [11,25].

### 2.4. Preparation and Characterization of Micelles

#### 2.4.1. Preparation of PLGA-*g*-PEI-*g*-CAGW Copolymer

The CAGW peptide grafted cationic copolymer of PLGA-*g*-PEI was prepared by two steps. In the first step, PLGA-*g*-PEI (0.20 g, 0.012mmol) and Et_3_N (28 μL) were dissolved in 5.0 mL of NMP in a dried three-necked flask. 5 mL diallyl-carbamyl chloride solution (0.045 mol/L) was added into this flask slowly for 2.0 h at 0 °C, and maintained for an additional 12 h. Then, the product was obtained after filtering, dialyzing, and lyophilization. In the second step, the product of the first step (25.0 mg) was dissolved in DMSO (5.0 mL). DMPA solution (1.0 mg DMPA in 1.0 mL DMSO) and CAGW solution (15 mg CAGW peptide in 1.0 mL DMSO) were subsequently added into the above solution. Then the mixed solution was treated by UV-light for 10 min. PLGA-*g*-PEI-*g*-CAGW copolymer was obtained by filtering, dialyzing, and lyophilization.

#### 2.4.2. Preparation of PLGA-*g*-PEI-*g*-CAGW Copolymer Micelles

PLGA-*g*-PEI-*g*-CAGW copolymer (0.125 g) was dissolved in 25 mL DMSO. 2 mL of this solution was added dropwise into 20 mL of phosphate buffer saline (PBS, pH = 7.4) in an Erlenmeyer flask. The PLGA-*g*-PEI-*g*-CAGW micelles were formed by this self-assembly method. 

The PLGA-*g*-PEI copolymer micelles were also prepared with an analogous method and used as a control.

#### 2.4.3. Characterization of PLGA-*g*-PEI-*g*-CAGW Copolymer Micelles

In order to verify the successful CAGW peptide linking onto the copolymer and determine the quantity of the peptide, we prepared a series of CAGW peptide solutions in PBS (pH = 7.4) to acquire the standard curve with concentrations varying from 0.1 to 1.0 mg/L. The fluorescence emission spectrum of PLGA-*g*-PEI and PLGA-*g*-PEI-*g*-CAGW copolymer micelles was measured by a Cary Eclipse (VARIAN) fluorescence spectrophotometer. We also measured the fluorescence emission spectrum of the mixed solution of PLGA-*g*-PEI and CAGW after dialysis. 

### 2.5. Preparation of PLGA-g-PEI-g-CAGW/pDNA and PLGA-g-PEI/pDNA Gene Complexes

pDNA (50 μg/mL) at different molar ratios of N/P (N/P = 2, 5, 10, 15, 20, 30, and 40, calculated from the weight of PEI and pDNA) was added dropwise into PLGA-*g*-PEI-*g*-CAGW or PLGA-*g*-PEI copolymer micelles (0.2 mg/mL) under stirring to obtain gene complexes, respectively. Before characterization and further experiments, these micelles were gently stirred and incubated for 30 min at room temperature.

### 2.6. Size and Zeta Potential of PLGA-g-PEI-g-CAGW/pDNA and PLGA-g-PEI/pDNA Gene Complexes

The hydrodynamic diameter (*D*_h_) and zeta potential of PLGA-*g*-PEI-*g*-CAGW and PLGA-*g*-PEI-*g*-CAGW/pDNA gene complexes with different N/P molar ratios (2, 5, 10, 20, 30, and 40) were determined by a dynamic light scattering instrument (DLS, Malvern Zetasizer NanoZS4700, Worcestershire, UK) equipped with vertically polarized light using a 633 nm argon-ion laser. The morphology of dried gene complexes at N/P of 30 was characterized by TEM JEM-2100F at 200 kV accelerating voltages.

### 2.7. Agarose Gel Electrophoresis

The pDNA condensation ability of the PLGA-*g*-PEI and PLGA-*g*-PEI-*g*-CAGW copolymer micelles was assessed by agarose gel electrophoresis. The PLGA-*g*-PEI/pDNA and PLGA-*g*-PEI-*g*-CAGW/pDNA gene complexes with various N/P molar ratios (N/P = 2, 5, 10, 20, 30 and 40) were prepared as described above. The gene complexes were loaded into the agarose gel (0.8%) containing 0.5 μg/mL ethidium bromide. The experimental procedure was similar to our previous work [10,25,27].

### 2.8. In Vitro Cytotoxicity of Copolymer Micelles and Gene Complexes

The cytotoxicity of PLGA-*g*-PEI, PLGA-*g*-PEI-*g*-CAGW copolymer micelles, and PLGA-*g*-PEI/pDNA and PLGA-*g*-PEI-*g*-CAGW/pDNA gene complexes were investigated by the MTT assay using PEI 10 kDa/pDNA gene complexes as a positive control. EA.hy926 cells (1.0 × 10^4^ cell/well) were seeded in 96-well plates, and cultured for 24 and 48 h as described in our previous work [25]. The relative cell viability (%) was calculated from the optical density at 24 and 48 h post-gene complex treatment [10,25].

### 2.9. In Vitro Transfection

EA.hy926 cells were seeded in 24-well plates at a density of 1.0 × 10^4^ cell/well, and cultured for 24 h as described in our previous work [25]. Before transfection experiments, cells were incubated in serum-free culture solution for 12 h. PLGA-*g*-PEI/pDNA or PLGA-*g*-PEI-*g*-CAGW/pDNA gene complexes at a N/P molar ratio of 30 were added into each well. The expression of green fluorescence protein (GFP) in the cells was observed under an inverted fluorescent microscope at 12 and 30 h time points.

### 2.10. Western Blot Assay

Western blot analysis was used to evaluate the expression of the ZNF580 gene. The experiment was carried out according to a previously reported method [25,37].

### 2.11. Wound Healing Assay 

The migration capability of EA.hy926 cells transfected by PLGA-*g*-PEI/pDNA, PLGA-*g*-PEI-*g*-CAGW/pDNA, and PEI 10 kDa/pDNA gene complexes at the N/P molar ratio of 30 was assessed by a wound healing assay [22,43]. The assay procedure was carried out according to our previous studies [10,25,27]. At 48 h post-gene complex treatment, EA.hy926 cells formed a cell monolayer, and then a liner wound was generated using a 200 μL sterile pipette tip at the middle of each well. D-hanks buffer (pH 7.4) was used to wash cellular debris. The migration process was monitored using an inverted microscope at different time points (0, 12, and 24 h). The relative recovery area (%) was calculated using the formula in our previous work [25].

### 2.12. Fluorescence-Activated Cell Sorting (FACS) Analysis

The transfected cells were washed twice with 0.01 mol/L PBS, and were then treated with 0.3 mL trypsin–EDTA solution for 30 s. After adding fresh growth medium, the cell suspensions were centrifuged at 1000 rpm for 10 min. The supernatant was removed, and the cells were re-suspended in 500 mL PBS. The transfection efficiency was determined by a flow cytometer (Beckman FC500, Beckman Coulter, CA, USA).

### 2.13. Statistical Analyses

All of experiments were performed at least three times and expressed as means ± SD. Statistical significance was analyzed using the Student’s *t*-test. *p* < 0.05 was considered to be statistically significant.

## 3. Results

### 3.1. Synthesis of PLGA-g-PEI and PLGA-g-PEI-g-CAGW Copolymers

**Figure 1:**
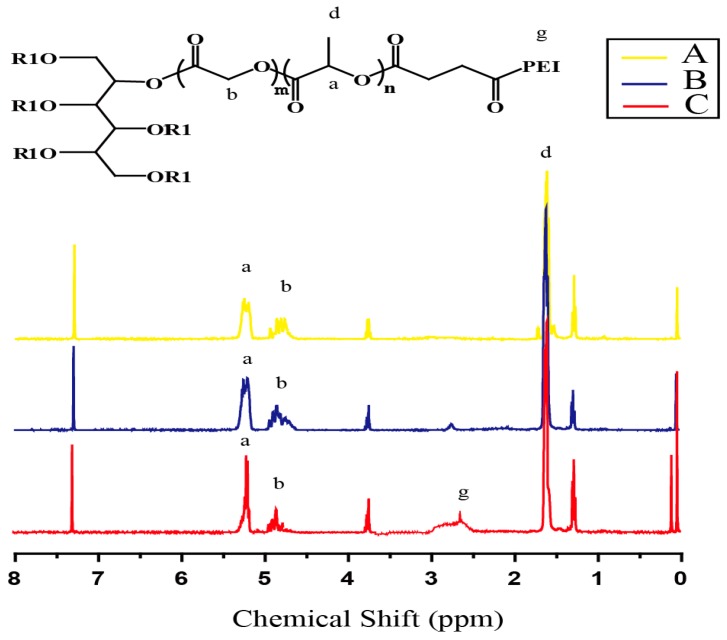
^1^H NMR spectra of PLGA-OH copolymer (**A**); PLGA-COOH copolymer (**B**) and PLGA-*g*-PEI copolymer (**C**) using CDCl_3_ as a solvent.

The cationic copolymer PLGA-*g*-PEI was synthesized in three steps. Firstly, a hexa-hydroxyl terminated star-shaped copolymer PLGA-OH was prepared by the ROP of d,l-LA and GA using sorbitol as an initiator and Sn(Oct)_2_ as a catalyst. The ^1^H NMR spectrum of PLGA-OH showed that the peaks at 5.22 ppm corresponded to d,l-LA residue (OC**H**(CH_3_)CO, 81.9 wt %), and the peak between 4.77 and 4.95 ppm corresponded to GA residue (OC**H**_2_CO, 18.1 wt %) (Figure 1A). Secondly, the terminal hydroxyl groups of PLGA-OH reacted with succinic anhydride to obtain the terminal carboxyl copolymer PLGA-COOH. This copolymer was confirmed by the additional peak at 2.69 ppm (OOCC**H**_2_C**H**_2_COOH) (Figure 1B). Thirdly, the cationic PLGA-*g*-PEI copolymer was prepared by the amidation reaction between PEI 10 kDa and carboxyl-terminated PLGA-COOH, and its ^1^H NMR spectrum showed a broad peak in the range of 2.68–3.0 ppm (NHC**H**_2_C**H**_2_) (Figure 1C). The molecular weight and polydispersity index (PDI) of the PLGA-OH copolymer were analyzed by GPC. *M*_n_ is 2.11 kDa, and *M*_w_ is 2.20 kDa with a PDI of 1.043. Unfortunately, the molecular weight of PLGA-*g*-PEI copolymer cannot be determined by GPC because of its low solubility in THF.

**Scheme 2:**
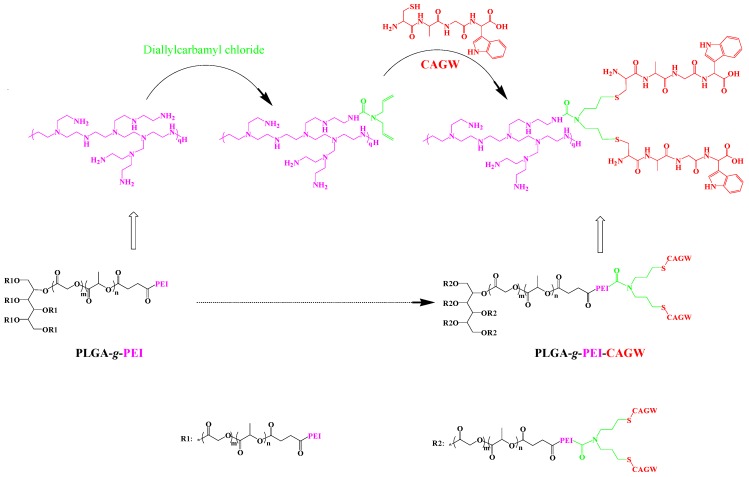
Synthesis route of PLGA-*g*-PEI-*g*-CAGW copolymer.

**Figure 2:**
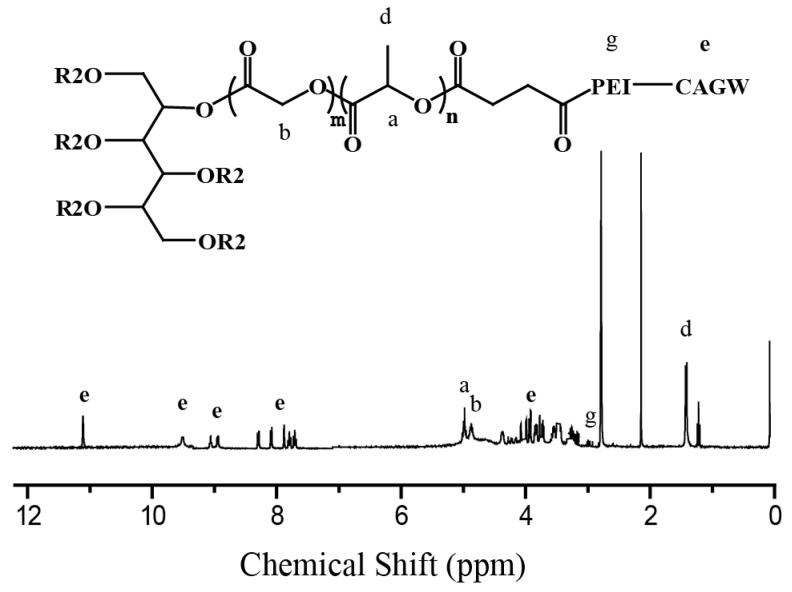
^1^H NMR spectrum of PLGA-*g*-PEI-*g*-CAGW copolymer using DMSO-d_6_ as a solvent.

**Figure 3:**
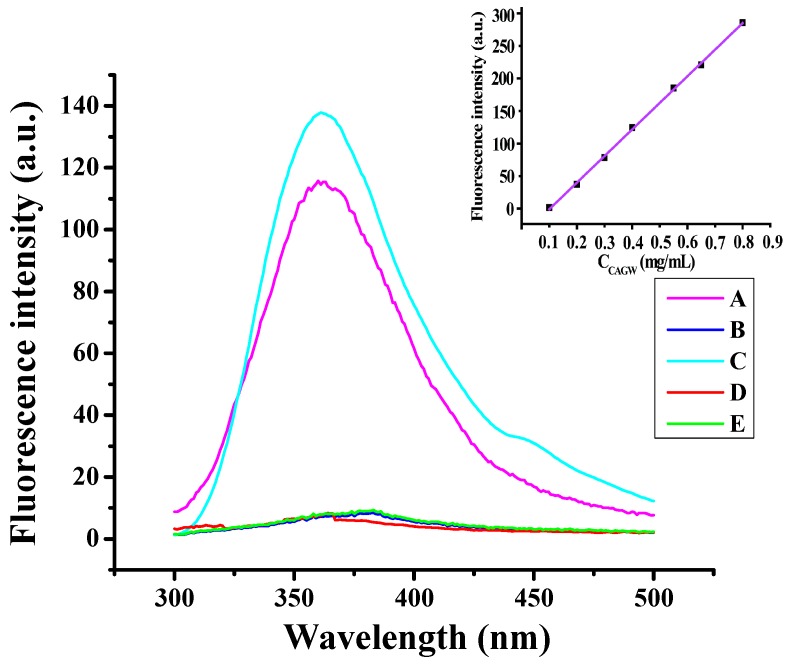
Fluorescence emission spectra of (**A**) PLGA-*g*-PEI-*g*-CAGW copolymer micelles; (**B**) PLGA-*g*-PEI copolymer micelles; (**C**) CAGW peptide aqueous solution (0.4 mg/mL); (**D**) the mixed solution of PLGA-*g*-PEI and CAGW peptide after dialysis; (**E**) the reaction mixture of PLGA-*g*-PEI, diallyl-carbamyl chloride, and CAGW peptide but without the application of UV-light for conjugation reaction after dialysis. The standard curve of the CAGW peptide is showed in the upper right.

In order to develop a gene carrier with high transfection efficiency, we linked the CAGW peptide onto the PLGA-*g*-PEI copolymer, because the CAGW peptide could be utilized to selectively promote EC adhesion [38,44]. The PLGA-*g*-PEI-*g*-CAGW copolymer was synthesized according to the route in Scheme 2. Its ^1^H NMR spectrum showed the peaks of CAGW (Figure 2), which proved the successful synthesis of the PLGA-*g*-PEI-*g*-CAGW copolymer. In this study, we used the CAGW peptide, which contains a CAG sequence and a tryptophan residue W (Scheme 2). The W residue can show a specific fluorescence emission spectrum at 350 nm when excited with 280 nm light [45]. As shown in Figure 3, the PLGA-*g*-PEI-*g*-CAGW copolymer micelles had a specific emission spectrum at 350 nm compared with PLGA-*g*-PEI copolymer micelles, indicating that the CAGW peptide has been successfully linked onto PLGA-*g*-PEI. To address whether the CAGW peptide was bound covalently or was just hydrophobically entrapped by PLGA-*g*-PEI, we carried out the synthesis process but did not apply the UV-light for the conjugation reaction. After dialysis of the reaction mixture, no emission spectrum at 350 nm was observed. This means that the dialysis can remove the free CAGW peptide from the reaction mixture, and the specific emission at 350 nm is due to grafting the CAGW peptide in the copolymer. According to the standard curve in the upper right of Figure 3 (*y* = 408.82·C − 41.92 (mg/mL), (*R*^2^ = 0.999)), the concentration of the CAG peptide in the PLGA-*g*-PEI-*g*-CAGW copolymer micelles was 0.385 mg/mL (the concentration of the PLGA-*g*-PEI-*g*-CAGW copolymer micelles was 15 mg/mL). 

### 3.2. Size and Zeta Potential of PLGA-g-PEI/pDNA and PLGA-g-PEI-g-CAGW/pDNA Gene Complexes

**Figure 4:**
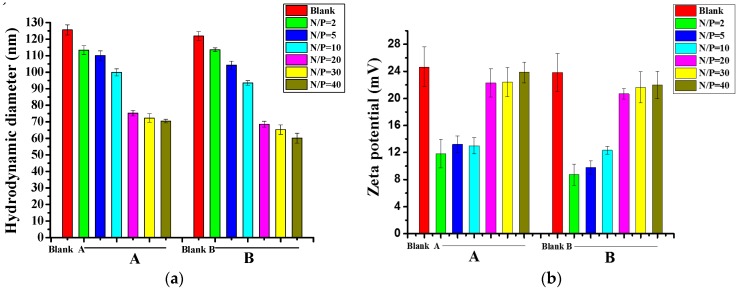
Hydrodynamic diameters and zeta potentials of gene complexes at various N/P molar ratios (2, 5, 10, 20, 30, and 40). (**a**) Hydrodynamic diameter; (**b**) Zeta potential. A: PLGA-PEI/pDNA gene complexes, B: PLGA-*g*-PEI-*g*-CAGW/pDNA gene complexes. Blank A: PLGA-*g*-PEI copolymer micelles, and Blank B: PLGA-*g*-PEI-*g*-CAGW copolymer micelles.

**Figure 5:**
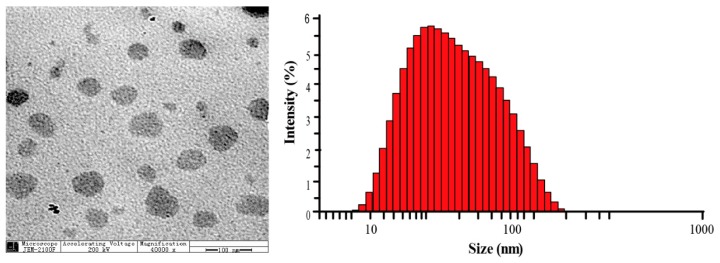
TEM image (**left**) and DLS histogram (**right**) of PLGA-*g*-PEI-*g*-CAGW/pDNA gene complexes at N/P = 30.

The hydrodynamic diameter (*D*_h_) and zeta potential of the PLGA-*g*-PEI/pDNA and PLGA-*g*-PEI-*g*-CAGW/pDNA gene complexes were determined using DLS. The size of the NPs is a key prerequisite for cellular uptake [46]. A small *D*_h_ value of the NPs (less than 200 nm) is beneficial for endocytosis [19,47]. As shown in Figure 4a, the *D*_h_ values of the PLGA-*g*-PEI and PLGA-*g*-PEI-*g*-CAGW micelles were 125.6 ± 3.0 nm and 121.8 ± 2.7 nm, respectively. After condensing with the pEGFP-ZNF580 plasmid, the gene complexes were smaller than the corresponding copolymer micelles. The gene complexes showed a large distribution; Figure 5 gives the PLGA-*g*-PEI-*g*-CAGW gene complexes as an example, and the others are summarized in Table 1. TEM images also demonstrated that the PLGA-*g*-PEI-*g*-CAGW/pDNA gene complexes Had irregular spherical shapes with different sizes, and the distribution was large (Figure 5). When micelles condensed with the pEGFP-ZNF580 plasmid, PEI blocks collapsed and resulted in gene complexes with smaller sizes. In addition, their diameters decreased with the increase of the N/P molar ratio because they evolved into more compact entities [46]. At an N/P of 30, the *D*_h_ values of the PLGA-*g*-PEI-*g*-CAGW/pDNA gene complexes were 65.3 ± 2.9 nm. The small size of the gene complexes is beneficial for cellular uptake [21]. 

As shown in Figure 4b, the NPs had relatively high zeta potential because of the grafted cationic PEI. With the increase of the N/P molar ratio, the zeta potential of the PLGA-*g*-PEI/pDNA gene complexes increased from 11.8 ± 2.1 mV to 23.9 ± 1.5 mV. After grafting with the CAGW peptide, the zeta potential was slightly lower. In addition, the zeta potential of the gene complexes also increased with the increasing N/P molar ratio. 

### 3.3. Agarose Gel Electrophoresis

**Figure 6:**
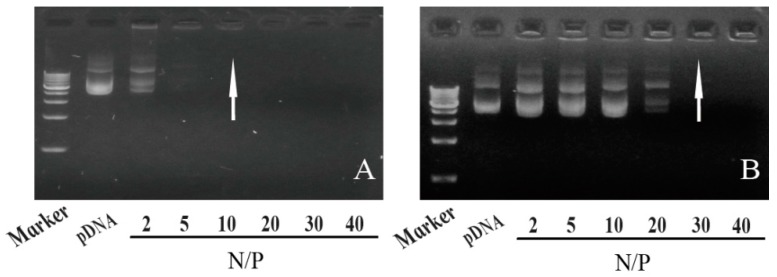
Agarose gel retardation assay of the PLGA-*g*-PEI/pDNA and PLGA-*g*-PEI-*g*-CAGW/pDNA gene complexes at different N/P molar ratios (2, 5, 10, 20, 30, and 40). (**A**) PLGA-*g*-PEI/pDNA gene complexes and (**B**) PLGA-*g*-PEI-*g*-CAGW/pDNA gene complexes.

Electrostatic interaction of the cationic copolymer with pDNA is an important prerequisite for gene carriers [22]. PLGA-*g*-PEI/pDNA and PLGA-*g*-PEI-*g*-CAGW/pDNA gene complexes could retard pDNA migration at N/P molar ratios of 10 and 30, respectively (Figure 6). Therefore, we selected N/P 30 in the following study.

### 3.4. In Vitro Cytotoxicity

PLGA-*g*-PEI and PLGA-*g*-PEI-*g*-CAGW cationic copolymer micelles could condense pDNA, and form gene complexes via a self-assembly method in PBS. These copolymers can assemble into NPs. When ECs were cultured in the medium with PLGA-*g*-PEI-*g*-CAGW/pDNA gene complexes, due to the active targeting ligands of the CAGW peptide on their surface, these gene complexes can be recognized and adhered specifically by the membrane receptor, and then entered into ECs by cytophagy (Scheme 1). After lysosome escape, gene complexes or pDNA could enter into the cell nucleus through path I or II [48,49]. Then, the proliferation and migration of ECs will be promoted by the overexpression of the ZNF580 protein.

**Figure 7:**
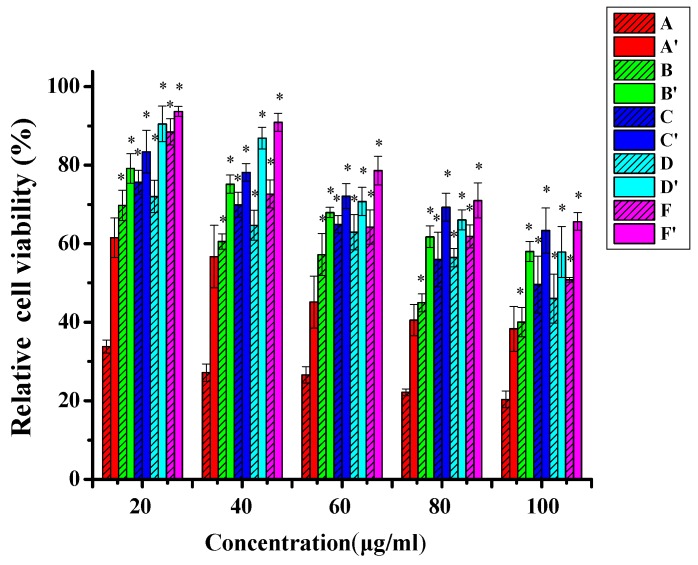
Relative cell viability of EA.hy926 cells after 24 h (with line) and 48 h (without line) of treatment with different concentrations of copolymer micelles and gene complexes at the N/P molar ratio of 30. PEI (10 kDa)/pDNA was used as a control group. (A,A’): cells treated by PEI (10 kDa)/pDNA gene complexes for 24 and 48 h; (B,B’): cells treated by PLGA-*g*-PEI copolymer micelles for 24 and 48 h; (C,C’): cells treated by PLGA-*g*-PEI/pDNA gene complexes for 24 and 48 h; (D,D’): cells treated by PLGA-*g*-PEI-CAGW copolymer micelles for 24 and 48 h; and (E,E’): cells treated by PLGA-*g*-PEI-*g*-CAGW/pDNA gene complexes for 24 and 48 h. Mean ± SD, *n* = 3, * *p* < 0.05 vs. control group.

It has been shown that too high positive charges could significantly cause cell death [37,50]. Safety and low cytotoxicity are the prerequisite for polymeric gene carrier in vivo applications. The toxicity of copolymers and gene complexes was evaluated by the MTT assay using PEI/pDNA as a control group. After the cells were treated with copolymer micelles or gene complexes for 24 and 48 h, the relative cell viabilities (%) were measured with the micelle concentrations ranging from 20 to 100 μg/mL. When the micelle concentration increased from 20 to 100 μg/mL, the relative cell viability of all groups decreased gradually. This phenomenon can be explained by the cytotoxicity of PEI in the copolymers. From Figure 7, we can find that the IC50 values (24 h) were about 80 and 100 μg/mL for the PLGA-*g*-PEI and PLGA-*g*-PEI-*g*-CAGW polymers, respectively.

**Figure 8:**
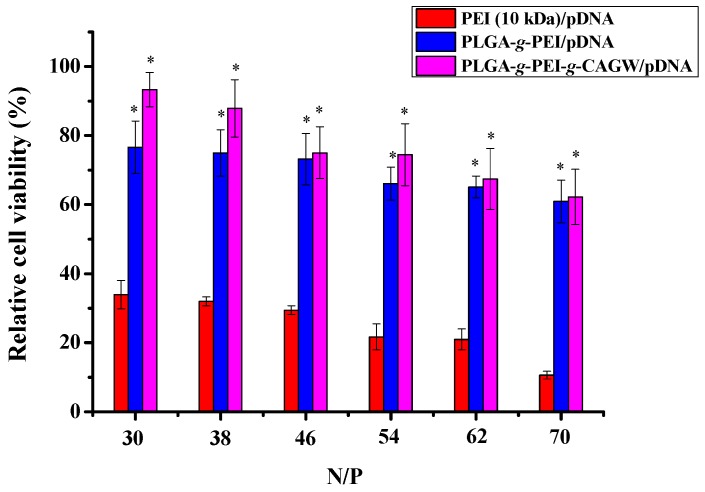
Relative cell viability with different N/P molar ratios at 24 h. EA.hy926 cells were treated by gene complexes with a concentration of 40 μg/mL. PEI (10 kDa)/pDNA was used as a control group. Mean ± SD, *n* = 3, * *p* < 0.05 vs. control group.

As shown in Figure 7, at the same concentration, the relative cell viability of the gene complex groups was much higher than the corresponding micelle groups. This phenomenon demonstrated that the neutralization between positive and negative charges could decrease the cytotoxicity [51]. Due to the grafting of the CAGW peptide, the positive charge of the PLGA-*g*-PEI-CAGW/pDNA gene complexes was relatively low. Thus, their cytotoxicity was lower than the PLGA-*g*-PEI/pDNA gene complexes at the same concentration. The positive charge of PEI is partially neutralized, which is beneficial in terms of low cytotoxicity [52]. In addition, the relative cell viability of the same group increased as the culture time increased from 24 to 48 h. When the N/P molar ratio increased from 30 to 70, the relative cell viability decreased slightly for both PLGA-*g*-PEI/pDNA and PLGA-*g*-PEI-CAGW/pDNA gene complex groups (Figure 8). These results demonstrated that the amount of PEI in the gene complexes mainly affected the cytotoxicity. 

### 3.5. In Vitro Transfection

**Figure 9:**
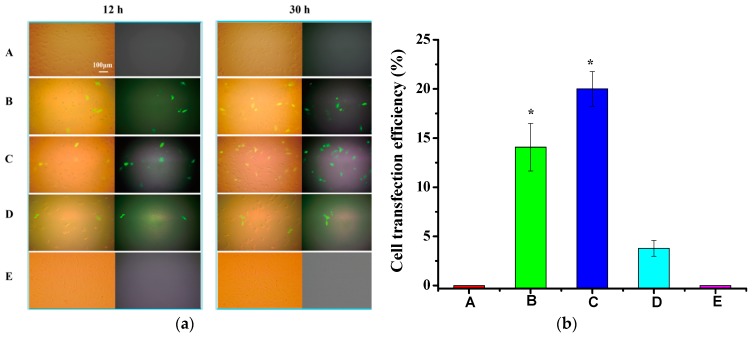
(**a**) Fluorescence images (right images) and corresponding bright-field images (left images) of EA.hy926 cells transfected for 12 h and 30 h. (**A**) Cells without any treatment as the negative control, (**B**) cells treated by PLGA-*g*-PEI/pDNA gene complexes, (**C**) cells treated by PLGA-*g*-PEI-*g*-CAGW/pDNA gene complexes, and (**D**) cells treated by PEI 10 kDa/pDNA gene complexes as the positive control, (**E**) SMCs treated by PLGA-*g*-PEI-*g*-CAGW/pDNA gene complexes. All of these experiments were performed with the N/P molar ratio of 30 and concentration of 40 μg/mL. Scale bar is 100 μm; (**b**) Transfection efficiency at 30 h. Mean ± SD, *n* = 3, * *p* < 0.05 vs. control group.

In order to determine the transfection efficiency of PLGA-*g*-PEI/pDNA and PLGA-*g*-PEI-*g*-CAGW/pDNA gene complexes in EA.hy926 cells, pEGFP-ZNF580 was used as a reporter gene, which is encoded with a GFP factor [53]. EA.hy926 cells were treated with these gene complexes in the culture solution. EA.hy926 cells were treated with pEGFP-ZNF580 as a negative control group, and cells were treated with PEI 10 kDa/pDNA as a positive control group. The expression of GFP in transfected cells was observed under an inverted fluorescent microscope. Figure 9a shows the fluorescence images (right images) and corresponding bright-field images (left images) of EA.hy926 cells without any treatment and treated by gene complexes at the N/P molar ratio of 30. A large amount of green fluorescence was highlighted in the PLGA-*g*-PEI/pDNA and PLGA-*g*-PEI-*g*-CAGW/pDNA groups, and cells treated with PLGA-*g*-PEI-*g*-CAGW/pDNA gene complexes were particularly prominent after an incubation of 30 h. In addition, the fluorescence intensity increased with the transfection time. The fluorescence intensity in the PLGA-*g*-PEI-CAGW/pDNA group was higher than that in the PLGA-*g*-PEI/pDNA group, and the fluorescence intensity of these two groups was higher than that of the positive control group. In order to further prove the selectivity of the CAGW peptide for ECs, SMCs were treated by the PLGA-*g*-PEI-*g*-CAGW/pDNA gene complexes. Almost no transfected SMCs were observed (Figure 9a(E)), which highlighted the targeting ability of the PLGA-*g*-PEI-*g*-CAGW/pDNA gene complexes to the ECs. The transfection efficiency of EA.hy926 cells mediated by gene complexes were evaluated by green fluorescence analysis. In the process of self-assembly, PEI 10 kDa chains were preferentially located at the surface of PLGA-*g*-PEI/pDNA and PLGA-*g*-PEI-*g*-CAGW/pDNA. Thus the high positive charge was a benefit for high transfection efficiency of the PLGA-*g*-PEI/pDNA and PLGA-*g*-PEI-*g*-CAGW/pDNA groups, compared with the PEI 10 kDa/pDNA group (Figure 9b). In particular, the transfection efficiency of the PLGA-*g*-PEI-*g*-CAG/pDNA group was much higher than the other groups, which may be attributed to the specific CAG peptide. These results proved that the CAG peptide is favorable for transfection efficiency, possibly due to the selective recognition of these gene complexes to ECs that is beneficial for cellular uptake. 

**Figure 10:**
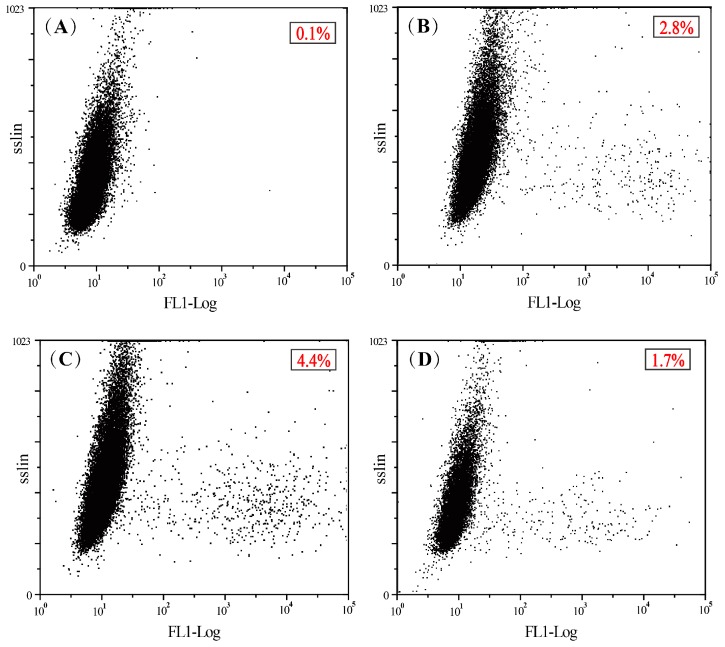
The transfection efficiency quantified by the measurement of GFP expression with flow cytometry. (**A**) Cells treated with naked Pdna; (**B**) cells treated with PLGA-*g*-PEI/pDNA gene complexes; (**C**) cells treated with PLGA-*g*-PEI-*g*-CAGW/pDNA gene complexes; (**D**) cells treated with PEI (10 kDa)/pDNA gene complexes.

The transfection efficiency of the PLGA-*g*-PEI/pDNA and PLGA-*g*-PEI-*g*-CAGW/pDNA gene complexes was also investigated by the cell flow cytometry method. Cells without any treatment were used as a negative control, and the cells treated with PEI 10 kDa/pDNA were used as a positive control. As shown in Figure 10, the PLGA-*g*-PEI/pDNA and PLGA-*g*-PEI-*g*-CAGW/pDNA gene complexes showed higher transfection efficiencies than the control group. Compared with the PLGA-*g*-PEI/pDNA gene complexes, the PLGA-*g*-PEI-*g*-CAGW/pDNA gene complexes exhibited more than a 1.57-fold higher transfection efficiency. These results also confirmed that the CAGW peptide can effectively transfect EA.hy926 cells.

### 3.6. Western Blot Analysis

**Figure 11:**
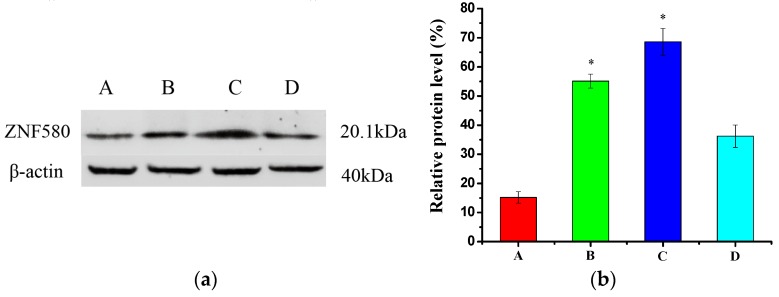
(**a**) Western blot analysis for ZNF580 protein expression in EA.hy926 cells transfected by different gene complexes after 48 h. (**b**) Relative protein level. (**A**) Cells without any treatment; (**B**) cells treated by PLGA-*g*-PEI/pDNA gene complexes; (**C**) cells treated by PLGA-*g*-PEI-*g*-CAGW/pDNA gene complexes and (**D**) cells treated with PEI 10 kDa/pDNA gene complexes. Mean ± SD, *n* = 3, * *p* < 0.05 vs. control group.

Western blot analysis was used to evaluate the expression of the ZNF580 gene. The ZNF580 protein level increased significantly in transfected EA.hy926 cells by the PLGA-*g*-PEI/pDNA and PLGA-*g*-PEI-*g*-CAGW/pDNA gene complexes. As shown in Figure 11a, after 48 h, the ZNF580 protein was overexpressed successfully in the PLGA-*g*-PEI-*g*-CAGW/pDNA gene complex group compared to the control groups of (A) and (D). The relative protein level was 68.6% in the transfected EA.hy926 cells in these gene complexes (group C), whereas 55.1% in the PLGA-*g*-PEI/pDNA group (B), and 36.2% in the PEI 10 kDa/pDNA group (D) (Figure 11b). These results demonstrated that the CAG peptide with its targeting ability benefits the expression of ZNF580 compared with the PLGA-*g*-PEI/pDNA. 

### 3.7. Wound Healing Assay

**Figure 12:**
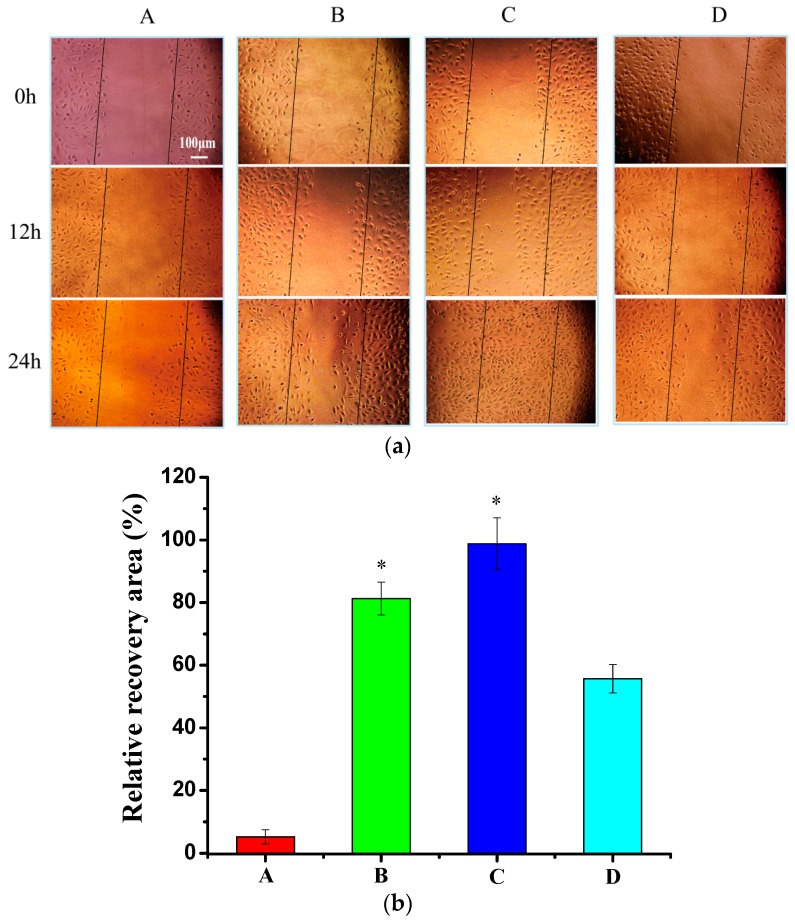
Wound healing assay of EA.hy926 cells at different time points (**a**) and relative recovery area (**b**) after 24 h calculated by Image-Pro Plus (2.0). (**A**) Cells without any treatment as the negative control; (**B**) cells treated with the PLGA-*g*-PEI/pDNA gene complexes; (**C**) cells treated with the PLGA-*g*-PEI-*g*-CAGW/pDNA gene complexes; (**D**) cells treated with the PEI (10 kDa)/pDNA gene complexes as a positive control. All of these experiments were conducted under with the N/P molar ratio of 30. Scale bar is 100 μm. Mean ± SD, *n* = 3, * *p* < 0.05 vs. control group.

The scratch wound assay was performed to evaluate the proliferation and migration of transfected cells [37]. EA.hy926 cells were treated with gene complexes at the N/P molar ratio of 30. Cells without any treatment were used as a negative control, and the cells treated with PEI 10 kDa/pDNA were used as a positive control. When the wound was produced for 24 h, the cells in the negative control group hardly migrated into the denuded area (Figure 12A). However, the cells treated with the PLGA-*g*-PEI/pDNA, PLGA-*g*-PEI-*g*-CAGW/pDNA, and PEI 10 kDa/pDNA gene complexes could migrate into the denuded area by different degrees (Figure 12B–D). The PLGA-*g*-PEI-*g*-CAGW/pDNA gene complex group showed the highest migration with a 98.8 ± 8.2% relative recovery area (Figure 12b). While the relative recovery area of the PLGA-*g*-PEI/pDNA group was 81.2 ± 2.6%, the value for the PEI 10 kDa/pDNA group was relative low (55.6 ± 4.5%), and the value for the negative control group was only 11.2 ± 2.2%. Due to the specific recognition of the CAG peptide to the integrin of ECs, the relative recovery area in the PLGA-*g*-PEI-*g*-CAGW/pDNA gene complex group was much higher than that in the PLGA-*g*-PEI/pDNA gene complex group. These results suggested that the PLGA-*g*-PEI-*g*-CAGW/pDNA gene complexes can promote the proliferation and migration of EA.hy926 cells.

## 4. Discussion

In recent years, gene technology has been used as an effective method to enhance EC proliferation and migration so as to promote rapid endothelialization. Peptides have been demonstrated to adhere to ECs effectively and selectively [23]. These technologies are effective strategies to promote the rapid endothelialization of biomaterials [54]. The growth of ECs on the surface of artificial blood vessels and the formation of endodermis through rapid endothelialization are beneficial for the promotion of hemocompatibility and the avoidance of restenosis and thrombosis. For gene technology, it is still a challenge to develop a safe and efficient gene delivery system, which should have high transfection efficiency and low cytotoxicity.

In our previous studies, some gene carriers have been designed and prepared from amphiphilic block copolymers based on cationic PEI, hydrophilic PEG, and biodegradable hydrophobic blocks of PLGA and PLMD [2,10,25,30]. These gene carriers combine the advantages of biodegradation and high transfection efficiency. It has been reported that the micelles formed from star-shaped copolymers show unique gene delivery effects [41]. In addition, star-shaped polymers exhibit low cytotoxicity and high transfection efficiency compared to linear polymers with the same molecular weight [20,55]. Herein, we prepared a kind of star-shaped copolymer PLGA with sorbitol as a skeleton and then an amphiphilic and biodegradable cationic copolymer of PLGA-*g*-PEI was subsequently synthesized in this paper. Hydrophobic PLGA segments provided the gene carriers with biodegradability and stability, while PEI (10 kDa) chains as the hydrophilic segments were located on the surface of the micelles [56,57]. In addition, the PLGA hydrophobic blocks served as a crosslinking point to provide the NPs with many PEI chains [58]. 

The ^1^H NMR and GPC were used to characterize the structure of the copolymers and the results indicated that the PLGA-*g*-PEI cationic copolymer was synthesized successfully. The CAGW peptide was covalently grafted on this copolymer via the thiol-ene click-reaction. The peptide content was determined by the characteristic fluorescence intensity at about the 350 nm emission wavelength of W (tryptophan) residue in PLGA-*g*-PEI-*g*-CAGW when excited by 280 nm light.

The PLGA-*g*-PEI and PLGA-*g*-PEI-*g*-CAGW copolymer micelles were formed by a self-assembly method in PBS (pH = 7.4). Both of them can condense the pEGFP-ZNF580 plasmid efficiently to form gene complexes. The shape of these gene complexes were irregular spheres with different sizes. These gene complexes had a suitable particle size and zeta potential for cellular uptake, measured by DLS. At the appropriate N/P molar ratio, NPs with small particle size and high zeta potential benefit from cellular uptake and gene transfection [59]. In addition, these gene complexes showed low cytotoxicity by the MTT tests. The wounding assay further suggested that the PLGA-*g*-PEI-*g*-CAGW/pDNA gene complexes can promote the proliferation and migration of EA.hy926 cells. The results of cell flow cytometry also demonstrated that the CAGW peptide benefits high transfection efficiency. In addition, these gene complexes are expected to more easily be transpired after the degradation of the ester bonds in the PLGA segments, which could subsequently reduce cytotoxicity. Noticeably, the CAG peptide has a special ability for adhesion of the ECs [31,32]. The gene carrier with the CAGW peptide could selectively enter into ECs by the receptor-ligand specific binding, and deliver the pEGFP-ZNF580 gene into the ECs. Compared with the PLGA-*g*-PEI gene carrier, the PLGA-*g*-PEI-*g*-CAGW gene carrier exhibited low cytotoxicity, high transfection efficiency, and high ZNF580 protein expression. These results indicated that the PLGA-*g*-PEI-*g*-CAGW can be applied to selectively deliver pDNA into the ECs and have great potential for rapid endothelialization.

## 5. Conclusions

PLGA-*g*-PEI-*g*-CAGW micelles as DNA carriers were studied with PLGA-*g*-PEI as a control in order to illustrate the contribution of the CAGW peptide grafted gene complexes in DNA delivery. The results indicated that the CAGW peptide conjugated gene complexes showed high efficiency of DNA delivery in vitro. Moreover, the PLGA-*g*-PEI-*g*-CAGW/pDNA gene complexes exhibited low cytotoxicity and enhanced transfection of EA.hy926 cells significantly better than the PLGA-*g*-PEI/pDNA gene complexes. Therefore, we believe that the PLGA-*g*-PEI-*g*-CAGW copolymer micelles are promising candidates for DNA delivery for applications of the endothelialization of biomaterial surfaces.

## Figures and Tables

**Table 1:** The size distribution of copolymer micelles (N/P = 0) and gene complexes with N/P molar ratios from 2 to 40.

N/P Molar Ratio	PLGA-*g*-PEI	PLGA-*g*-PEI-*g*-CAGW
0	0.460	0.454
2	0.349	0.306
5	0.411	0.550
10	0.406	0.362
20	0.286	0.267
30	0.400	0.332
40	0.373	0.792
